# Cell hierarchies in colorectal cancer: focus on APC and BRAF

**DOI:** 10.18632/oncoscience.225

**Published:** 2015-08-31

**Authors:** Markus Morkel, Pamela Riemer

**Affiliations:** Charité Universitätsmedizin Berlin, Institute of Pathology, Laboratory of Molecular Tumour Pathology and Systems Biology, Berlin, Germany

**Keywords:** Wnt Signaling, Beta-Catenin, MAPK signaling, intestinal stem cells, mouse models

The Wnt-APC-β-Catenin and RAS-RAF-MAPK signaling pathways serve to maintain homeostasis in the normal intestinal epithelium. This state is characterized by an equilibrium between stemness, proliferation, differentiation and cell death [1]. Recent studies by our group [2] and by Dow *et al*. [3] elucidate how oncogenes and tumor suppressors influence cell hierarchies during colorectal cancer (CRC) formation in the mouse and highlight the potential of this information for future approaches to cancer therapy.

A majority of CRCs are initiated by APC mutations promoting Wnt/β-Catenin activity and stem cell fate [4]. As we described already in 2012, reversible transgenic expression of oncogenic β-Catenin can switch mouse intestinal epithelium between normal and adenomatous cell hierarchies [5]. For these experiments, we used organotypic cell cultures maintaining stem, proliferative and differentiated cells. β-Catenin promoted a universal capacity for self-renewal and expression of cancer stem cell markers, similar to cultures derived from APC^−/−^ adenoma. Withdrawal of oncogenic β-Catenin re-established normal cell hierarchies driven by only few stem cells in the crypts. A new publication by Dow *et al*. [3] shows that reversible knockdown of APC in the intestine of mice establishes adenomatous growth *in vivo*, while restoration of APC results in reversion to normal cell hierarchies and tumor regression. When the authors combined reversible APC knockdown with conditional gain- and loss-of-function alleles for the KRAS oncogene and the p53 tumor suppressor, respectively, invasive colon carcinoma developed. Significantly, restoration of APC resulted in complete remission and reverted colon cancer tissue to normal intestinal epithelium even in the presence of oncogenic KRAS and absence of p53. These results demonstrate a surprising ability of intestinal epithelium to regain homeostasis.

In contrast to Wnt/β-Catenin, the roles of MAPK activity in the control of intestinal cell hierarchies are not well established. EGFR-RAS-RAF-MAPK transduces crucial proliferative cues in the intestinal crypt. Oncogenic KRAS has a tumor-promoting role in conventional adenoma initiated by loss of APC, while oncogenic BRAF can initiate another type of CRC precursor, termed serrated adenoma, independently of APC [4]. We recently found that transgenic expression of oncogenic BRAF caused intestinal serration, but also abrogated maintenance of stem cells in the mouse intestine, which collectively converted to proliferative progenitors [2]. How can BRAF act as a CRC initiator despite its negative effect on stem cell support? The answer comes from a publication dissecting multi-step CRC development after oncogenic BRAF knock-in in the mouse [6]: activation of BRAF resulted in generalized intestinal hyperplasia with a reduced stem cell pool, and only progressive foci displayed activating mutations in the Wnt/β-Catenin pathway, nuclear β-Catenin and increased gene expression of Wnt targets and stem cell markers. This suggests that increased β-Catenin activity can supplement the stem cell pool after oncogenic activation of BRAF. Indeed, we found that activation of β-Catenin partially protected intestinal tissue from the deleterious effects of BRAF overexpression *in vitro* and *in vivo* [2]. Taken together, these results give insight into the mechanisms controlling cell hierarchies in CRC, and suggest that the Wnt/β-Catenin and MAPK signaling pathways must be activated in a coordinate manner to allow for CRC initiation and progression.

**Figure 1:**
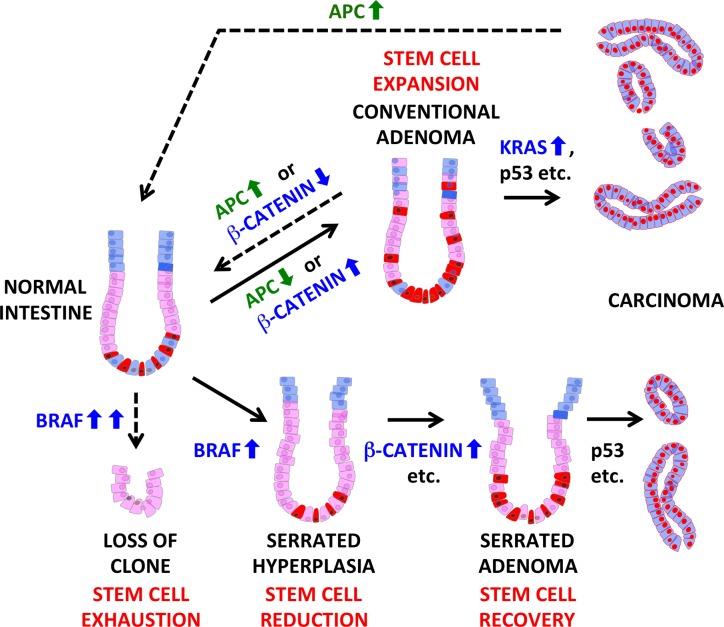
Balancing of the stem cell pool in mouse models of intestinal cancer development Top: conventional progression pathway via knock-down/loss of APC or expression of stabilized β-Catenin; Bottom: serrated progression pathway initiated by activation of BRAF. Dashed lines indicate non-malignant endpoints, either by tissue reversion (restoration of APC or inactivation of stabilized β-Catenin) or stem cell exhaustion (BRAF hyperactivation). Mutations in key oncogenes and tumor suppressors are given in blue and green, respectively. Changes in the stem cell pool are indicated in red. Code for cell types: stem cells (red); proliferative progenitors (pink); differentiated cells (blue); cancer cells (red-blue).

How can constraints on cell hierarchies be exploited for novel cancer therapies? Dow *et al*. argue that cancer remission upon APC restoration provides a strong case for the Wnt signaling pathway as a therapeutic target [4]. Panels of organotypic cultures derived from human CRC have recently become available [7]. Thus, restoration of APC in human canceroids could provide further evidence for the validity of the concept proposed by Dow and colleagues. Therapies targeting the EGFR-MAPK axis are already in clinical use and in clinical trials, such as EGFR-inhibitors and combinatorial inhibition within the extended MAPK network [4]. Future strategies could take into account vulnerabilities of the stem cell pool towards MAPK signals in certain types of CRC.
